# Diagnosis and treatment of Guillain‐Barré syndrome during the Zika virus epidemic in Brazil: A national survey study

**DOI:** 10.1111/jns.12358

**Published:** 2019-12-03

**Authors:** Sonja E. Leonhard, Rodrigo M. Conde, Francisco de Assis Aquino Gondim, Bart C. Jacobs

**Affiliations:** ^1^ Department of Neurology Erasmus University Medical Center Rotterdam The Netherlands; ^2^ Ribeirão Preto Medical School University of São Paulo (FMRP‐USP) Ribeirão Preto São Paulo Brazil; ^3^ Department of Internal Medicine, Neurology Division Federal University of Ceará Fortaleza Ceará Brazil; ^4^ Department of Immunology Erasmus University Medical Center Rotterdam the Netherlands

**Keywords:** clinical practice, Guillain‐Barré syndrome, management, survey, Zika virus

## Abstract

The Zika virus (ZIKV) epidemic in Brazil in 2015‐2016 was followed by an increase in the incidence of patients with Guillain‐Barré syndrome (GBS). With this national survey study, we aimed to gain a better understanding of how neurologists in Brazil are currently diagnosing and treating patients with GBS, and how this increase in incidence has impacted the management of the disease. The questionnaire consisted of 52 questions covering: personal profile of the neurologist, practice of managing GBS during and outside of the ZIKV epidemic, and limitations in managing GBS. All 3264 neurologists that were member of the Brazilian Academy of Neurology at the time of the study were invited to participate. The questionnaire was fully answered by 171 (5%) neurologists. Sixty‐one percent of neurologists noticed an increase in patients with GBS during the ZIKV epidemic, and 30% experienced an increase in problems in managing GBS during this time. The most important limitations in the diagnosis and management of GBS included the availability of nerve conduction studies (NCS), beds in the Intensive Care Unit (ICU) and referral to rehabilitation centers. Most neurologists did not use a protocol for treating patients with GBS and the treatment practice varied. Increasing availability of NCS and beds in the ICU and rehabilitation centers, and the implementation of (inter)national guidelines, are critical in supporting Brazilian neurologist in their management of GBS, and are especially important in preparing for future outbreaks.

## 1 INTRODUCTION

Guillain‐Barré syndrome (GBS) is the most common acute paralytic neuropathy worldwide, with a global incidence of ~1‐2 per 100 000 person‐years.^1^ GBS typically presents as progressive weakness and sensory signs, starting in the distal legs and progressing to the arms and facial muscles.^2^ Disease progression is rapid and often severe, with ~20% of patients requiring mechanical ventilation due to involvement of respiratory muscles.^2^ Treatment for GBS generally consists of multidisciplinary supportive medical care and immunotherapy. Both intravenously administered immunoglobulin (IVIg) and plasma exchange are proven effective therapies for GBS.^3^

**Figure 1:**
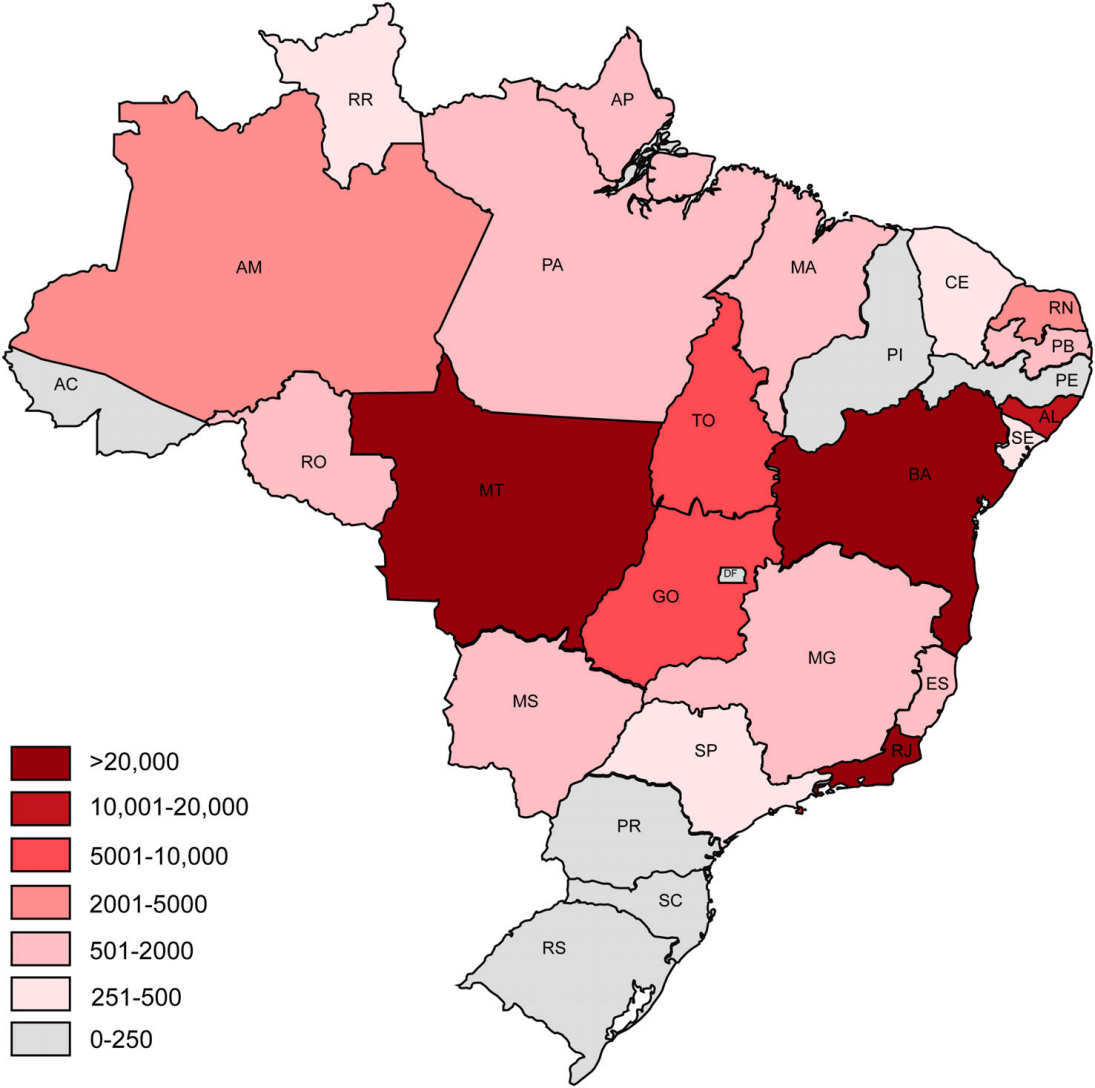
Number of reported suspected Zika virus cases per state in Brazil, 2016. This figure displays the number of reported suspected ZIKV cases in 2016 per state in Brazil, as published by the Brazilian Ministry of Health in in 2017.^15^ Not all cases were laboratory confirmed, and other arbovirus infections, were often not excluded. Brazil is divided into 27 states and five regions. The five regions are: North (AC, Acre; AP, Amapá; AM, Amazonas; PA, Pará; RO, Rondônia; RR, Roraima; TO, Tocatins), Northeast (AL, Alagoas; BA, Bahía; CE, Ceará; MA, Maranhão; PB, Paraíba; PE, Pernambuco; PI, Piauí; RN, Rio Grande do Norte; SE, Sergipe), Center‐West (GO, Goiás; MT, Mato Grosso; MS, Mato Grosso do Sul; DF, Distrito Federal), Southeast (ES, Espírito Santo; MG, Minas Gerais; RJ, Rio de Janeiro; SP, São Paulo), and South (PR, Paraná; RS, Rio Grande do Sul; SC, Santa Catarina)

GBS is an immune‐mediated neuropathy and in most cases presumed to be triggered by specific types of infections.^2^ Several pathogens have been associated with GBS in case‐control studies.^4^ Most recently, infection with Zika virus (ZIKV) was associated with GBS, when incidence peaked during the outbreaks of ZIKV in Latin America in 2015‐2016.^5^, ^6^ Brazil was one of the countries most severely affected by the ZIKV epidemic, with ~370 000 cumulative ZIKV cases (suspected or confirmed) reported by the World Health Organization and Ministry of Health between December 2015 and January 2018.^7^ The actual incidence is likely to be even higher, as cases may have gone underreported, considering ZIKV usually causes a mild and uncomplicated or subclinical infection. The number of reported cases and the incidence in Brazil was highest in the Northeast, Southeast, and Center‐West regions (Figure 1).^8^

It is unknown how neurologists in Brazil are currently managing GBS and if the ZIKV epidemic has affected the diagnosis and treatment of GBS patients. Apart from a protocol mainly directed to guide clinicians in decisions on therapy, there are currently no detailed national Brazilian guidelines for the diagnosis and management of GBS, and at the time of the survey no international guidelines were available.^9^ This may complicate the management of the syndrome, especially because clinical presentation and disease progression can differ extensively between patients and specific diagnostic or prognostic markers for GBS are not yet available. Furthermore, it is unknown if neurologists experience limitations in the availability of diagnostic tools, treatment, or care for GBS, and if the increase of GBS patients during the ZIKV epidemic affected such availability.

To gain a better understanding of the current clinical practice in the management of GBS in Brazil and the impact of the ZIKV epidemic, we have conducted a national survey study among Brazilian neurologists. With this survey, we identified limitations in the diagnosis and management of GBS in Brazil, both during and outside of outbreak periods. This information can help in developing strategies to improve the clinical practice of GBS in Brazil, and to prepare for future outbreaks of ZIKV or other pathogens that may trigger GBS.

## 2 METHODS

### 2.1 Ethical approval

This study was approved by the Ethical Review Board of the Ribeirão Preto Medical School of the University of São Paulo (FMRP‐USP) and the National Ethical Research Commission of Brazil (Comissão Nacional de Ética em Pesquisa, CONEP).

### 2.2 Questionnaire

The questionnaire was developed by S.E.L., R.M.C., F.d.A.A.G., and B.C.J., and was reviewed for consistency, readability, completeness, and question sequencing by three independent GBS experts. Questions were drafted in English and translated to Brazilian Portuguese by an annotated translation agency. The questionnaire consists of 52 questions, with 41 multiple choice and 11 open‐ended questions, covering several topics, including: personal profile of the neurologist, their practice of managing GBS during and outside of the ZIKV epidemic and limitations they experience in managing GBS. The questionnaire was distributed via an online platform (Limesurvey) that guarantees anonymous and secure data storage and is approved by the Erasmus University Medical Center for the conduction of survey studies.

### 2.3 Study population

All the neurologists that were member of the Brazilian Academy of Neurology (Academia Brasileira de Neurologia) were approached through the Academy to participate in the survey study. They were contacted via e‐mail, containing a link to the online Limesurvey platform. The first invitation was sent in February 2019 and participants had a total of 70 days to answer the questionnaire. Five reminders were sent during that time.

### 2.4 Analysis

Statistical analysis of multiple‐choice questions was done using IBM SPSS Statistics 25 and included descriptive statistics and comparative analyses (Chi square, Fisher's exact test). Two researchers (S.E.L. and R.M.C) independently grouped open‐ended questions into categories. Discrepancies in interpretation were discussed to reach consensus.

## 3 RESULTS

A total of 3264 neurologists were member of the Brazilian Academy of Neurology at the start of the survey and were invited to participate in the study. Of this group, 254 (8%) answered the questionnaire, and of these responses, 171 (5%) were complete. For the analysis, only fully completed questionnaires were used.

### 3.1 Profile of the neurologists

**Figure 2:**
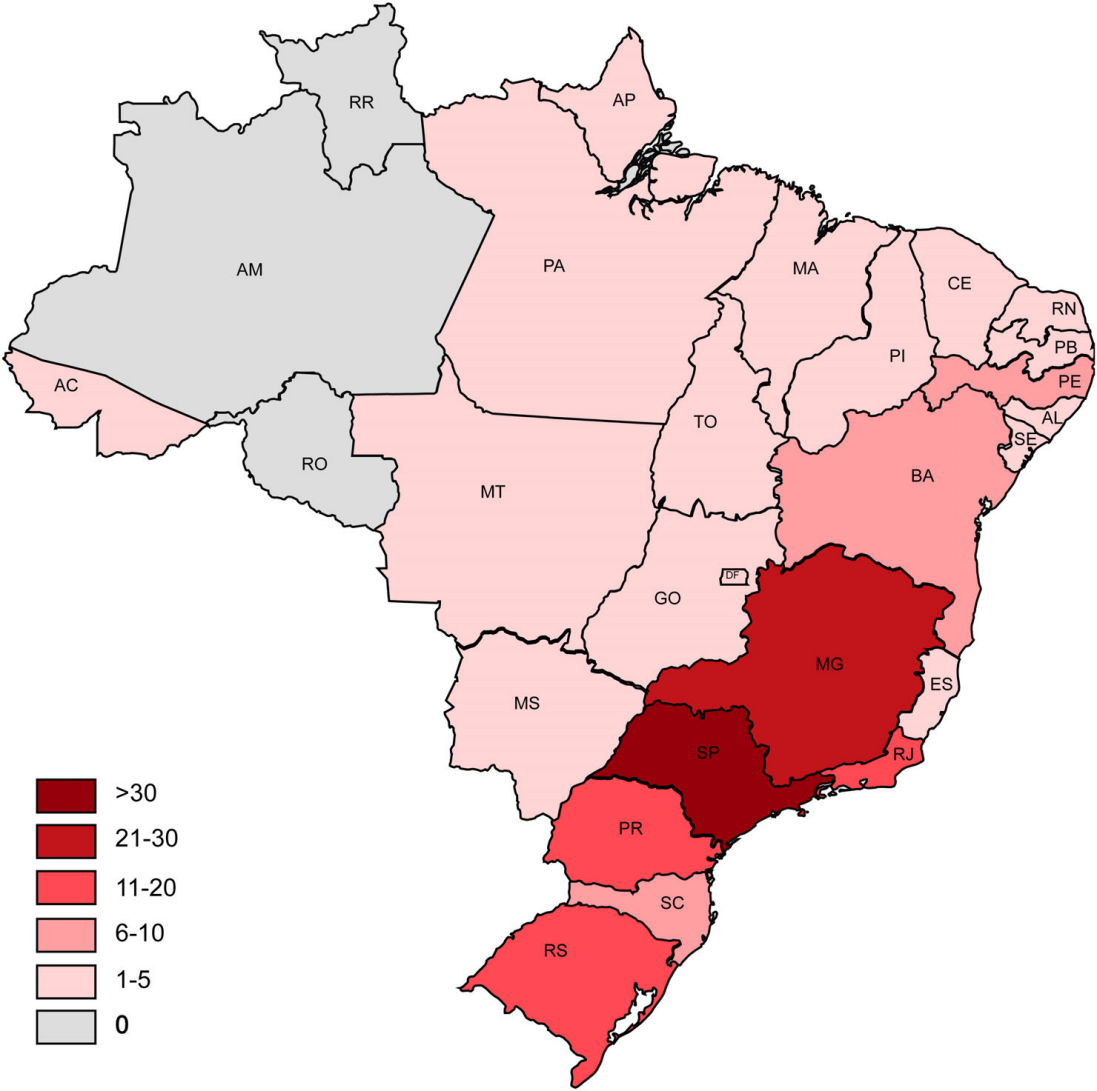
Geographic distribution of responding neurologists (N = 171). This figure displays the number of responding neurologists per state in Brazil

The profile of the responding neurologists is described in Table 1. The responders are well‐varied regarding age, field of interest, and employment in the private vs public sector. The majority of neurologists work in one hospital (49%), some in two (36%) and a few in three (15%). Most neurologists work in hospitals in the city of São Paulo (11%), Rio de Janeiro (9%), Ribeirão Preto (6%), Belo Horizonte (6%), and Curitiba (5%). Corresponding to this, responders most frequently work in the Southeast region of Brazil (54%), followed by the South (18%), Northeast (17%), Center‐Wester (8%) and North (3%) (Figure 2).

**Table 1:** Profile of responding neurologists (N = 171)

Age	40 (34‐49)
Male: Female (ratio)	96:75 (1.28)
Years practicing as neurologist	10 (5‐20)
Field of specialization or interest	
General neurology	103 (64)
Neuromuscular disorders	60 (37)
Neuro‐immunology	42 (26)
Vascular disorders	31 (19)
Movement disorders	30 (19)
Epilepsy	27 (17)
Neurodegenerative	26 (16)
Pediatric neurology	12 (7)
Neuro‐oncology	5 (3)
Number of newly diagnosed GBS cases per year	
0	4 (2)
1‐5	98 (57)
6‐10	50 (29)
11‐20	14 (8)
>20	5 (3)
Affiliation in public and/or private hospital	
Only public	49/156 (31)
Only private	64/156 (41)
Public and private	43/156 (28)

*Note*: Data are displayed as n/N (%), median (IQR) or n:n (ratio). For questions with multiple answer formats, percentages do not add up to 100.

Abbreviation: GBS, Guillain‐Barré syndrome.

### 3.2 Diagnosis

The clinical practice in diagnosis and treatment of GBS is shown in Table 2. Criteria that were used for diagnosing GBS included the criteria developed by the National Institute of Neurologic Diseases and Stroke (NINDS) (1978, revised in 1990) and by the Brighton Collaboration (2010).^10^, ^11^, ^12^ Fifteen percent of the neurologists indicated they used other criteria or no specific criteria.

**Table 2:** Clinical practice of GBS diagnosis and treatment

Diagnostic criteria used	
NINDS	71 (42)
Brighton Collaboration	98 (58)
Other or no specific/published criteria^a^	29 (15)
Treatment protocol used	64/168 (38)
Treatment indication	
All GBS patients are treated	81/171 (48)
Specific treatment indication^b^	
Rapid disease progression	80/90 (89)
Inability to walk independently (any distance)	69/90 (77)
Inability to walk independently for 10 m	13/90 (14)
(Imminent) respiratory insufficiency	76/90 (84)
Swallowing dysfunction	72/90 (80)
Severe autonomic dysfunction	72/90 (80)
Standard treatment (first line)	
IVIg	162 (95)
PE	3 (2)
IVIG and IV corticosteroids (combination)	4 (2)
IVIg or PE	2 (1)
Alternative treatment^c^ (second line)	28/54 (52)
PE	12/29 (41)
IV corticosteroids	6/29 (21)
Other^d^	7/29 (24)
No response to treatment	
Switch to other treatment	106 (62)
Repeat treatment	67 (39)
No additional treatment	13 (8)
Start corticosteroids	7 (4)
Other^e^	7 (4)
Indication ICU admission^b^	
Inability to walk independently (for any distance)	42 (25)
Inability to walk independently for ≥10 m	8 (5)
(Imminent) respiratory insufficiency	163 (95)
Rapid disease progression	142 (83)
Swallowing dysfunction	117 (68)
Severe autonomic dysfunction	147 (86)
Other^f^	3 (2)

*Note*: Data are displayed as n/N (%) or median (IQR). For questions with multiple answer formats, percentages do not add up to 100.

Abbreviations: NINDS, National Institute of Neurologic Diseases and Stroke,^11^, ^12^ Brighton, Brighton collaboration criteria;^10^ IVIg, intravenous immunoglobulin; IV, intravenous; PE, plasma‐exchange; ICU, Intensive Care Unit.

a Protocolo Clínico e Diretrizes Terapêuticas (n = 5), American Academy of Neurology Guideline on immunotherapy for GBS (n = 1), BMJ Best Practice guideline for GBS (n = 1).

b Multiple answers were possible. Answer option “Inability to walk for any distance” was considered mutually exclusive for “Inability to walk for 10m.”.

c Only neurologists that indicated that the preferred treatment was not always available were asked this question.

d PE or corticosteroids (n = 3), PE or IVIg (n = 1), referral to other hospital (n = 1), non‐pharmaceutical support (n = 2).

e Start (intensive) rehabilitation (n = 2), depends on the individual patient (n = 2), re‐evaluation of diagnosis (n = 3).

f All acute GBS cases (n = 2), clinical complications (n = 1).

**Figure 3:**
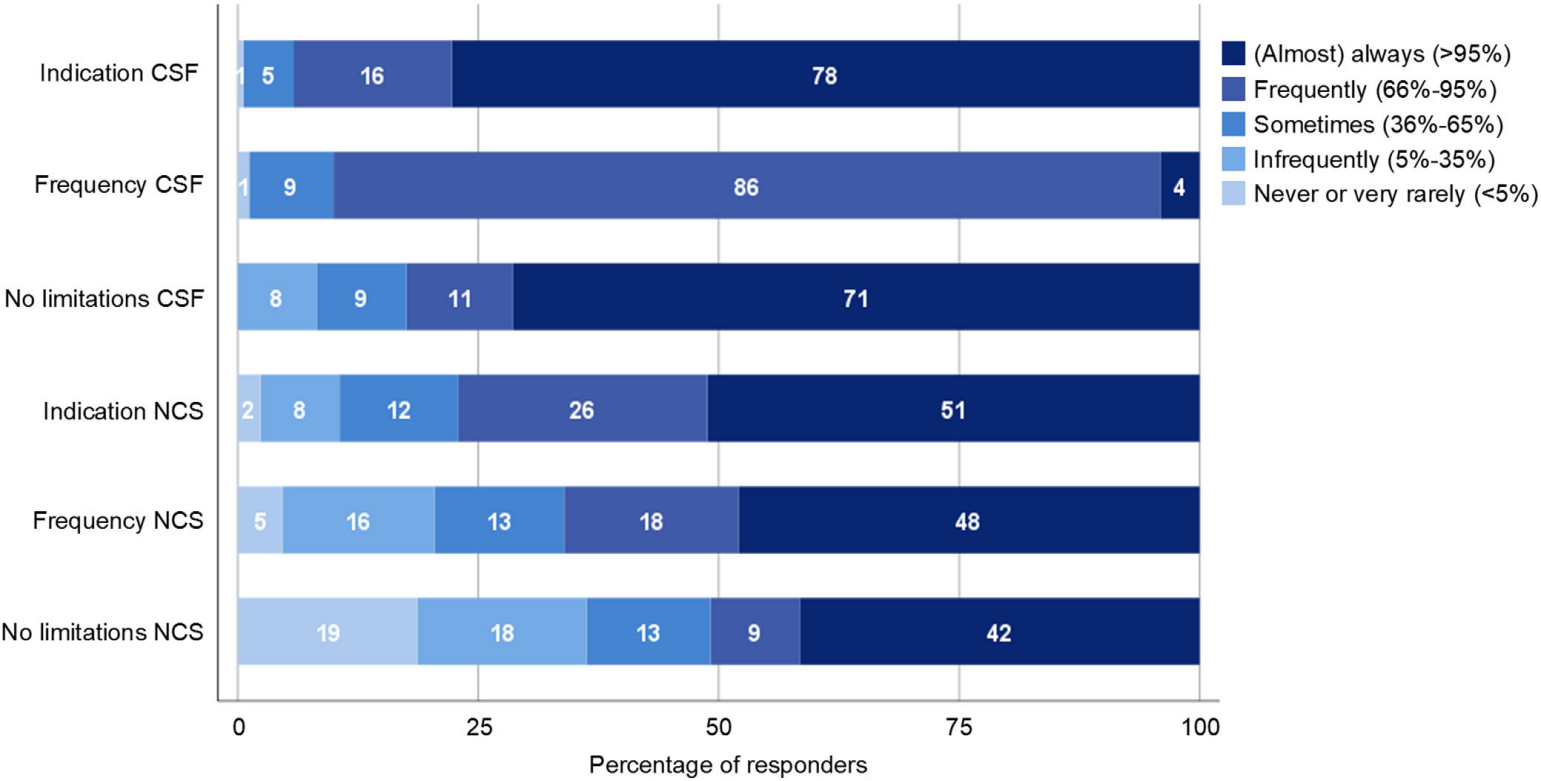
Diagnosis: indication, frequency, and availability of CSF and NCS. This figure displays how often neurologists considered CSF or NCS to be indicated in the diagnosis of GBS (“indication”), how often they used these diagnostics tools (“frequency”), and how often they encountered limitations in using these diagnostics (“no limitations”)

According to most of the neurologists, cerebrospinal fluid (CSF) testing was (almost) always indicated for diagnosing GBS, but only 4% (almost) always tested CSF in suspected GBS cases. This discrepancy may be explained in part by practical limitations in the opportunity to examine CSF, which were experienced sometimes or frequently by 17% of neurologists (Figure 3). These limitations included the availability of laboratory testing (71%), personnel (33%), equipment (17%), and high costs of the procedure (17%).

Nerve conduction studies (NCS) were available at the hospital of 57% of neurologists. Fifteen percent of the neurologists that indicated NCS were not available at their hospital did not or could not always refer the patient to a dedicated clinic. NCS were frequently or (almost) always indicated in the diagnosis of GBS according to 77% of neurologists, but fewer neurologists (66%) frequently or (almost) always made use of this diagnostic tool (Figure 3). This may be explained by limitations in NCS, that were frequently or (almost) always present in 36% of the responders, and included limited availability of personnel (65%), equipment (57%), high costs of the procedure (24%), and transportation issues (7%). Any limitations in CSF and NCS were experienced more often by neurologists working in public hospitals vs those only working in private hospitals, and those working in the Northeast and Center‐West vs other regions, although this was only significantly different for CSF examination (*P* = .04, respectively *P* < .001).

### 3.3 Treatment and care

Most of the neurologists did not use a specific protocol to treat GBS patients. Of the 64 neurologists who indicated to use a specific protocol, only seven provided details of this protocol. These protocols included the Protocolo Clínico e Diretrizes Terapêuticas (PCDT), an expert opinion protocol that is approved by the Ministry of Health of Brazil; the American Academy of Neurology (AAN) Guideline on immunotherapy for GBS; and the BMJ Best Practice guideline on GBS.^9^

When asked what they consider to be the best treatment for GBS, 60% of neurologists answered that IVIg and plasmapheresis are equally effective, followed by 35% who considered IVIg to be the best treatment. However, IVIg was the standard treatment for GBS in the vast majority of responders. According to 48% of neurologists, starting treatment is indicated in all GBS patients, regardless of clinical presentation, severity or progression. When asked what the maximum time period was after the start of neurologic symptoms that they would consider starting treatment in GBS patients, most indicated to start treatment within 1 month (49%) or 2 weeks (23%), and 11% of neurologists did not have any restrictions.

**Figure 4:**
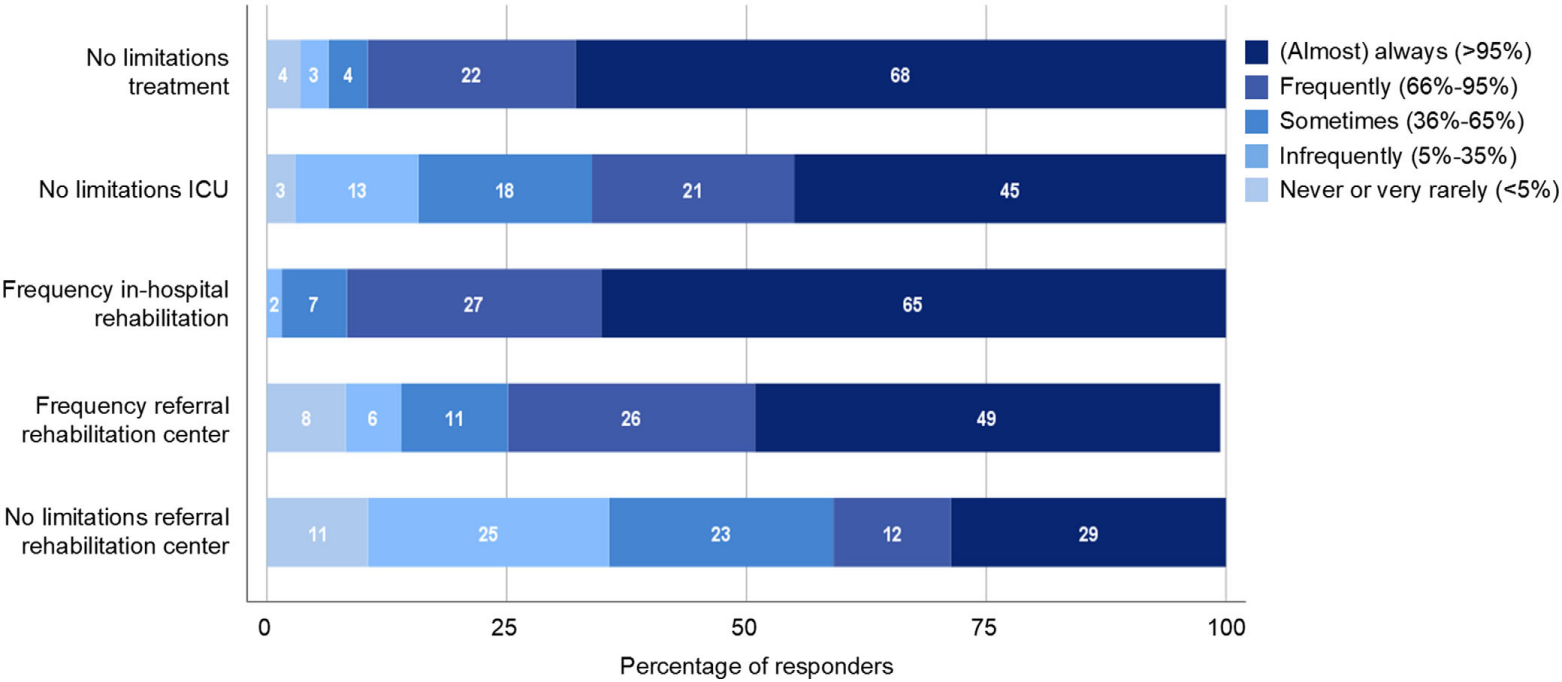
Management: frequency and availability of treatment, ICU and rehabilitation. This figure shows how often neurologists encountered limitations in the availability of the best treatment for GBS, ICU admission, and referral to a rehabilitation unit (“no limitations”), and how often patients received in‐hospital rehabilitation and were referred to a rehabilitation unit (“frequency”). For the variable “frequency referral to rehabilitation center,” one responder used the answer option “other”

Although the preferred treatment was (almost) always available for most responders, for 11% treatment was available only sometimes, infrequently, very rarely or even never (Figure 4). When the preferred treatment was not available, alternative treatment most often consisted of plasmapheresis or IV corticosteroids. Reasons for limited availability of the preferred treatment included high costs (55%), limited access to IVIg within the public health system (33%) and staff or logistics‐related issues (38%).

If a patient does not respond to treatment, 51% of neurologists would switch to another treatment (eg, plasmapheresis if first treatment was IVIg or vice‐versa), 27% would repeat the same treatment, and for 12% both repeating and switching therapy were an option. Of the responders that would repeat treatment, 48% would repeat for a maximum of two times, and 12% had no restrictions in how often they would repeat treatment. Neurologists who indicated to have expertise in neuro‐immunology or neuromuscular diseases were more likely to repeat treatment (*P* = .02), and less likely to switch treatment (*P* < .001) compared to other neurologists. Treatment practice did not significantly differ between neurologists who had more experience (>5 years) or who saw more (≥5) GBS patients yearly.

Although an ICU was available in the hospital of 96% of neurologists, 55% experienced limitations in transferring GBS patients to the ICU (Figure 4). A limited amount of beds at the ICU was the main problem, indicated by 98% of the responders.

A rehabilitation program was available in the hospital of 77% of the responders. If present, the program included physical therapy (100%), speech therapy (86%), psychosocial support (60%), and occupational therapy (39%). Referral to a rehabilitation unit at discharge was common, although a quarter of neurologists indicated that this was done only sometimes, infrequently, or never or very rarely. Limitations in referring patients to a rehabilitation unit were experienced by the majority of responders, of which 36% experienced this frequently or (almost) always (Figure 4). The most important limitations were a lack of available beds (54%), no rehabilitation center in the region (25%), and limited accessibility of rehabilitation for patients in the public health sector (28%), including delay due to administrative procedures.

Neurologists working in the public sector more frequently experienced any limitations in intensive care (*P* = .03) and referral to a rehabilitation unit (*P* = .03) compared to those only working in the private sector. Any limitations in treatment and ICU availability were more frequent in northern states, although this did not statistically differ between regions.

### 3.4 GBS during the ZIKV epidemic

**Figure 5:**
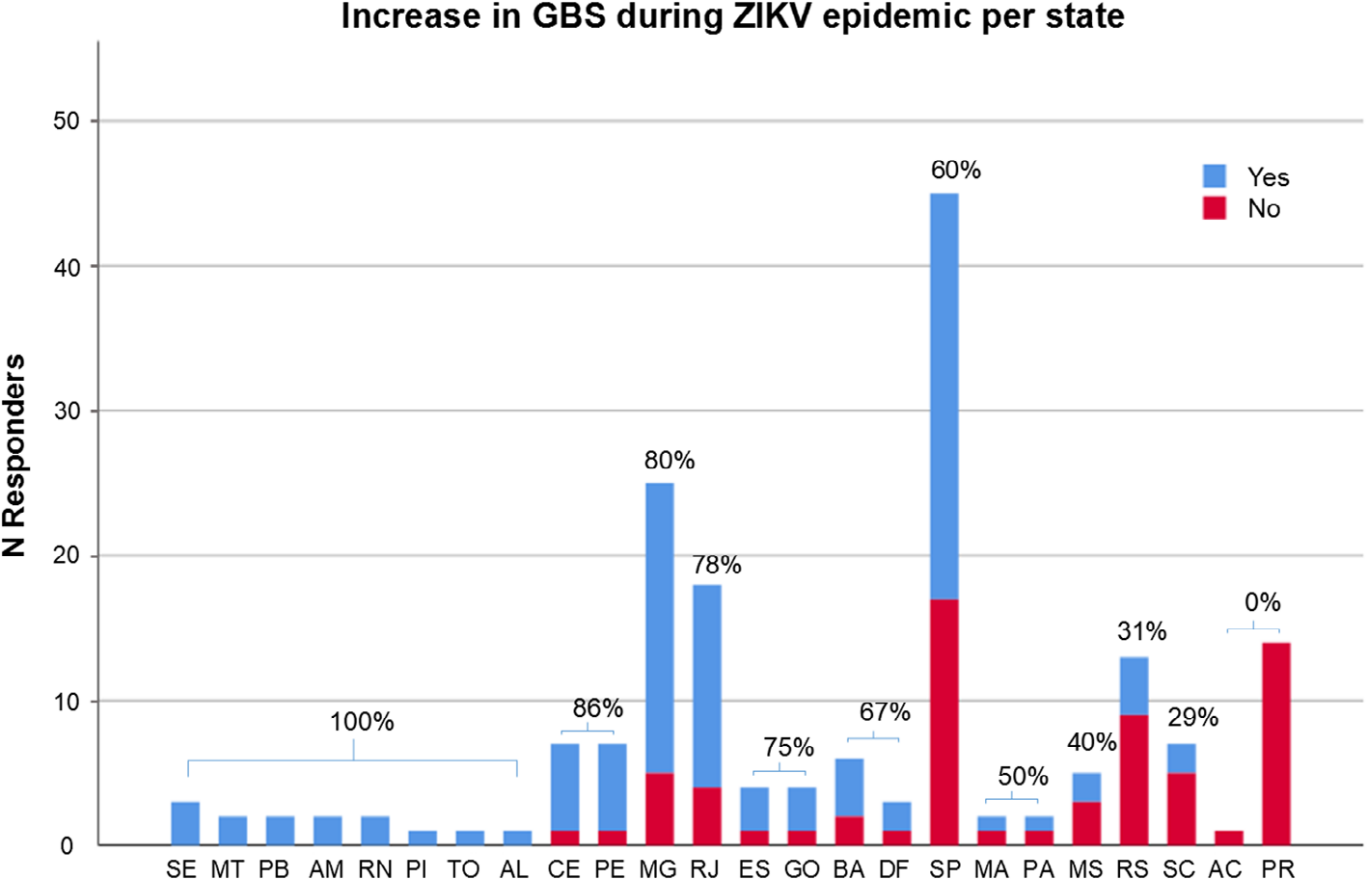
Increase in GBS during ZIKV epidemic displayed per state. Increase in GBS patients during ZIKV epidemic in Brazil (2015‐2016) as perceived by the responding neurologists displayed as number of responders per state, with percentage perceiving increase per state

During the ZIKV epidemic in Brazil, 61% of neurologists observed an increase in admissions of patients with GBS in their hospital and 30% of these neurologists experienced an increase in problems in the management of GBS patients. These increased problems included limitations in the opportunity to perform NCS (68%) and CSF examination (27%), availability of beds at the hospital (32%) and the ICU (59%), and availability of treatment (41%). An increase in GBS patients during the ZIKV epidemic was observed most often by neurologists working in the Northeast of Brazil, and an increase in patients or problems in the management were less frequent in the southern regions (*P* < .05) (Figure 5).

At the time of answering the questionnaire, 59% of neurologists tested for ZIKV in (selected) patients with GBS. Of these neurologists, 74% tested for ZIKV PCR, 73% for ZIKV IgM and only three neurologists indicated to use a plaque‐reduction neutralization test.

## 4 DISCUSSION

Neurologists in Brazil often experience limitations in the opportunity to conduct NCS and to refer to the ICU and to rehabilitation centers when confronted with patients with GBS. Most neurologists saw an increase in GBS patients during the ZIKV epidemic, and one‐third of these neurologists also experienced an increase in problems in managing GBS patients during that time. The treatment practice of GBS of neurologists in Brazil is comparable to treatment practice found in other regions, and indicates that international guidelines are necessary, especially to help deciding when to start and when to repeat treatment in patients with GBS.^13^

Limitations in the diagnosis or treatment of GBS were experienced frequently. Any limitations in NCS were experienced by 60%, in ICU care by 55%, and in referring patients to a rehabilitation center by ~30% of neurologists. Especially the lack of availability of sufficient intensive care for GBS patients is worrying, as this may directly affect morbidity and even mortality of these patients. Limitations were generally more frequently experienced by neurologists working in the Northeast and Center‐West of Brazil, which corresponds to a lower socioeconomic status in these regions, as reflected by regional contribution to the gross domestic product.^14^ Both neurologists working at the public and private sector experienced these limitations, but they were more frequent in the public health system. So although the public health system in Brazil (Sistema Único de Saúde) provides universal health coverage for all inhabitants of the country, including access to adequate treatment and care for GBS patients, our data indicate that in practice, access is not guaranteed and often delayed, and that a lack of availability of equipment, treatment, and beds in the ICU and rehabilitation centers occur frequently.

During the ZIKV outbreak in Brazil, about 60% of neurologists experienced an increased frequency of GBS patients admitted to their hospitals, about one‐third of whom also experienced an increase in problems managing GBS. Increase in patients with GBS was experienced most frequently in the North, Northeast and Center‐West of Brazil, reflecting the areas that were subject to the highest incidence of ZIKV during the outbreak in 2016.^15^ Limitations in availability of NCS and ICU admission were again the biggest issues. Furthermore, only 3% of neurologists indicated to use plaque‐reduction neutralization test in ZIKV, which is a laboratory test that helps to differentiate between a recent ZIKV and dengue virus infection. Due to cross‐reactivity, ZIKV and dengue virus can be difficult to tell apart based on serology, and implementation of this test can be crucial in correctly diagnosing patients with ZIKV, especially when PCR results are negative.^16^, ^17^ Lack of usage of this test suggests that identification of ZIKV‐related GBS cases may not be optimal.

Most neurologists do not use a specific protocol for treating GBS patients, and in some situations the treatment practice varies between neurologists or deviates from available evidence from treatment trials. First, of the 29 neurologists that use an alternative treatment when the preferred treatment (IVIg or plasmapheresis) for GBS is not available, about 20% use IV corticosteroids, although studies have proven that corticosteroids are not effective in treating GBS and may even have a negative effect on outcome.^3^ Second, about half of neurologists switch treatment and about a third repeat treatment in patients that do not (sufficiently) respond to therapy, although no evidence exists that this is effective. Third, about half of neurologists start treatment in all patients with GBS, regardless of their clinical condition, although effectivity of plasmapheresis and IVIg has not been sufficiently studied in patients still able to walk.^18^, ^19^ This treatment practice is also common outside of Brazil, and can likely be explained by the lack of evidence and the absence of international guidelines for treatment in these situations.^13^

This study has several limitations. First, the percentage of responding neurologists was limited, and may be biased toward specific neurologists, for instance those working in the neuromuscular field, in academic hospitals, or in certain regions. Second, the results presented reflect the estimates and opinions of neurologists, that may deviate from the actual practice.

## 5 CONCLUSION

Increasing international migration of humans and vectors pose threats of new epidemics of ZIKV or other arbovirus infections, potentially related to GBS, resulting eventual upsurge of GBS incidence in the near future.^20^ Our survey identified several critical limitations in the current practice of managing GBS in Brazil and can direct the development of strategies to improve this. Most importantly, the lack of availability of NCS, intensive care management and rehabilitation of GBS should guide redistribution of available funding from the Brazilian government or (inter)national nonprofit organizations. Furthermore, treatment practice of GBS is variable and few neurologists use guidelines in treating patients with GBS. Increasing the visibility of the existing national expert opinion protocol for the management of GBS (PCDT), or implementation of a recently published expert‐based international guideline for the management of GBS may help to provide such guidance.^9^, ^21^

## CONFLICT OF INTEREST

S.E.L. declares no conflict of interest. R.M.C. declares no conflict of interest. F.d.A.A.G. declares that he received honoraria from Baxter and CSL Behring to be speaker in IVIg treatment. B.C.J. has received funding from Annexon Biosciences, Baxter, CSL Behring, Hansa Biopharma and Grifols.

## AUTHOR CONTRIBUTIONS

S.E.L. and B.C.J. conceptualized the study. All authors designed the questionnaire. S.E.L. and R.M.C. structured the database for analysis and S.E.L. performed all the analyses. S.E.L. wrote the first draft of the manuscript. All authors contributed to the discussion of article content and edited the manuscript before submission.
